# School entrance examinations as a small-scale data source for health monitoring of children using obesity as an example

**DOI:** 10.25646/11435

**Published:** 2023-06-14

**Authors:** Charlotte Kühnelt, Anne Starker, Gianni Varnaccia, Anja Schienkiewitz

**Affiliations:** 1 Robert Koch Institute, Berlin Department of Epidemiology and Health Monitoring; 2 Formerly Robert Koch Institute, Berlin Department of Epidemiology and Health Monitoring

**Keywords:** SMALL-SCALE DATA, SCHOOL ENTRANCE EXAMINATIONS, OBESITY, CHILDREN, INDICATORS

## Abstract

**Background:**

In the scope of the nationwide obligatory school entrance examinations (SEE), a standardised assessment of the preparedness for school of preschool children takes place in the federal states of Germany. For this purpose, height and weight of the children are determined. These data are available in aggregated form at county level, but are not yet being regularly compiled and processed at national level for use in policy and research.

**Methods:**

In a pilot project, the indexing and merging of SEE data from 2015–2019 was tested in collaboration with six federal states. This was done using obesity prevalence at the time of the school entrance examination. In addition, prevalences were linked to small-scale indicators on settlement structure and sociodemographics from public databases, differences in obesity prevalence at county level were identified, and correlations to regional influencing factors were visualised.

**Results:**

It was feasible to merge SEE data from the federal states with little effort. The majority of the selected indicators were freely available in public databases. In an interactive, easily comprehensible and user-friendly Tableau dashboard for visualising the SEE data, it can be seen that obesity prevalences differ significantly between counties that are similar in terms of settlement structure or sociodemographics.

**Conclusions:**

Providing federal state SEE data and linking them to small-scale indicators enables region-based analyses and cross-state comparisons of similar counties and provides a data basis for continuous monitoring of the prevalence of obesity in early childhood.

## 1. Introduction

Small-scale data, e.g. at county or district level, are of central importance for the planning, implementation and evaluation of prevention and health promotion measures as well as for the provision of health care at federal, state and county levels. In terms of the ‘Public Health Action Cycle’ [1], aggregated data for small-scale analyses are an essential requirement for identifying existing needs in order to make health policy decisions based on actual evidence. However, few data sources are available that provide small-scale, meaningful data on the population health status or on health related risk and protective factors [2].

One such data source are the nationwide mandatory school entrance examinations (SEE), whose potential has not yet been fully used. The SEE are conducted nationwide by the Child and Adolescent Health Services (KJGD) at county level or at district level in the city states. This involves a regular, standardised examination of body weight and height of children aged four to seven years, as well as of other parameters. It is the aim of the SEE to assess children’s preparedness for school and to identify deficits in their development. In addition to assessing social-emotional, cognitive and motor skills, the SEE also include a physical and medical examination, in which body height and weight are measured. The height and weight data and the classification of whether the child examined is overweight or obese are reported back to the respective parents and are thus mainly used for personal medical purposes. Some federal states regularly publish SEE data in the form of reports [3–6], dashboards [7, 8] or tables [9, 10]. In 2007, SEE data from the federal states on the prevalence of overweight and obesity in children starting school were researched nationwide and published for the first time [11]. A compilation and processing of the data for policy and (public health) research as well as for national health reporting has not yet taken place.

Overweight and, in particular, obesity in children and adolescents are associated with long-term adverse health outcomes such as type 2 diabetes and cardiovascular diseases [12]. In Germany, about 9% of children between the ages of three and six years are overweight, and about 2% are obese [13]. School enrolment age is considered to be a crucial time slot for the manifestation of obesity in children and adolescents [14]. The study ‘German Health Interview and Examination Survey for Children and Adolescents (KiGGS)’ was able to show that more than half of the children with overweight/obesity aged two to six years remain overweight or obese as adolescents. There are significant regional differences in the prevalence of obesity and its influencing factors [15]: According to a 2019 survey of federal states, between 8.1% and 13.0% of the children in SEE are overweight and between 2.8% and 6.0% of children are obese [16]. Within the framework of health monitoring, health risks, such as overweight and obesity, which are susceptible to appropriate action, are of central importance in the evaluation of the impact of interventions at population-level and the regular reassessment of the need for action.

The AdiRaum pilot project at the Robert Koch Institute (RKI) investigates the potential of SEE data for the description of obesity prevalences at small-scale level.

One project goal is to index the data from the SEE at county or district level and make it usable for nationwide health monitoring. The nationwide health monitoring’s task is to continuously monitor the development of disease incidences as well as the health and risk behaviour in Germany [17]. In addition, trends and changes in the health status are to be identified and analysed in relation to previous or future prevention measures.

Another aim of AdiRaum is to link the obesity prevalences available at small-scale level to additional geospatial information from publicly accessible sources and to present them visually. This is meant to enable the identification of similarities and differences in the prevalence of obesity at the time of the SEE at county level and to show possible correlations between the prevalence and regional influencing factors.

This paper describes the conceptual and practical procedure as well as the visualisation of the results using the prevalence of obesity at the time of the SEE for exemplary purposes.

## 2. Methods

**Figure 1:**
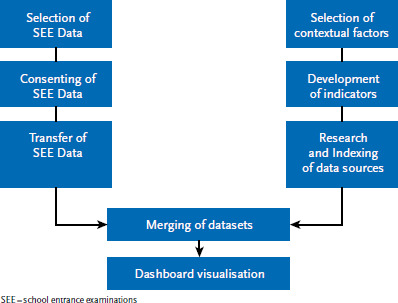
Work steps of AdiRaum for the development of small-scale data from school entrance examinations (SEE) using obesity as an example Source: Own diagram

AdiRaum was conducted from May to December 2022. Due to limited resources, the pilot project was tested with six federal states. This involved representatives from the health reporting departments of the states of Bavaria, Berlin, Brandenburg, Lower Saxony, North Rhine-Westphalia (NRW), and Saxony, as well as RKI staff. In the project, the merging of the small-scale SEE data as well as the linkage of this data to additional geospatial information was planned and implemented jointly with the federal states (see Figure 1).

### 2.1 Data from the school entrance examinations

The implementation of the SEE is regulated in federal state-specific manner. In order to be able to assess the extent to which the SEE data of the federal states are nevertheless comparable, similarities and differences in methodology and content of the SEE were identified in cooperation with the representatives of the federal states. This concerned, for example, the respective specifications for the recording of age, height and weight or examination periods and cut-off dates for school enrolment. The representatives of the health reporting departments of the participating federal states agreed to submit aggregated data on overweight and obesity to the RKI. The definition of overweight and obesity was based on the month-specific age- and sex-specific 90th and 97th percentiles according to Kromeyer-Hauschild [18]. For the pilot project, the years of enrolment 2015 to 2019 and, exclusively, data from initial examinations were selected. The reporting year corresponded to the year of enrolment. Counties merged during 2015 to 2019 due to regional reorganisation were adjusted to the most current county structure for the respective years. For uniform data transfer to the RKI, an Excel template was created in which the average age in months at the time of the SEE, the number of children examined and the number of children with overweight and obesity were to be entered for each county (rural county, independent city or district), year of age and sex.

### 2.2 Selection of contextual factors and indicators

#### Step 1: Selection of contextual factors

In AdiRaum, contextual factors were defined to be health-influencing factors that represent a person’s background [19]. On the one hand, contextual factors were taken into consideration that are relevant for the identification of counties that are similar in terms of settlement structure or sociodemographics. On the other hand, contextual factors that are relevant regarding the prevention of obesity at preschool age were considered. The selection was based on the topics that were identified in a systematic literature review in the AdiMon project (Population-wide monitoring of factors that influence childhood obesity) [20]. Additional factors relevant for the federal health reporting for the group of zero- to six-year-olds, such as from the context of child day care, were discussed and selected with the participating federal states.

#### Step 2: Selection of indicators

Indicators that are relevant for identifying counties with similar settlement structure or sociodemographics and for the prevention of obesity at preschool age were identified and selected through literature search. This was also based on the existing indicator system of AdiMon.

#### Step 3: Research and indexing of data sources

A review of databases providing the indicators free of charge and easily accessible was conducted in order to collect the data for the agreed indicators. If no information was available in the common, freely accessible databases, such as the Regionaldatenbank [21] or the INKAR database [22], the data were requested directly from data holders, such as regional sports confederations or Statistical Offices. If multiple similar indicators were available for a contextual factor or if several data sources were available for an indicator, the indicators were prioritised according to the Z.W.E.R.G. criteria (German abbreviation for importance, economic efficiency, simplicity, timeliness, accuracy) [23] and the characteristics periodicity, representativeness, regionalisability applied earlier in AdiMon. The corresponding data were then extracted from the available data sources or, occasionally, commissioned as special evaluations.

### 2.3 Visualisation of the results

For visualisation of the obesity prevalences, an interactive dashboard was created using TableauTM software, which enables the comparison of prevalences at county level (rural county, independent city or district) and differentiation by geospatial contextual factors. Dashboards can include various illustrations such as charts and maps and represent interactive, easily comprehensible, and user-friendly alternatives to tables. Besides free access and the ability to share the dashboard via a link, the requirement was that the data could be updated and expanded.

## 3. Results

### 3.1 Data from the school entrance examinations

Participating federal states submitted SEE data on overweight and obesity from 216 counties; no data were available from two counties only. One federal state was able to provide analysable data for 2015, one federal state for 2015 to 2018, and four federal states for 2015 to 2019. Four out of six federal states submitted data for children aged four to seven, two states for five- to six-year-olds. Data from the federal states were merged into one data set. Accordingly, a total of 1,627,949 children were examined. Considering the federal states’ confidentiality rules, 96.5% (1,570,568 children) with complete data were included in the analyses.

The confidentiality rules of the federal states aim to prevent re-identification of the examined children included and are applied when the number of examined children, the number of children with overweight or the number of children with obesity was below a state-specific defined threshold. In these cases, no data were reported to the RKI, but the number of children was reported in aggregated form: <3, <5, ≤5, or <10 depending on the federal state. If the number of examined children was below the respective threshold of the confidentiality rule, the children were excluded from the data set. If the confidentiality rule concerned the number of overweight or obese children, a value was imputed into the corresponding cell using the midpoint of the respective confidentiality rule, for example, if <5, a value of 2.5 was imputed.

The dashboard shows differences in the prevalence of obesity among children of school enrolment-age at county level. At county, independent city, and district levels, significant differences in prevalence were evident not only within but also between federal states. In the year of enrolment 2015 the average prevalence for all federal states was 4.0%, ranging from a lowest observed prevalence in one county of 0.5% to a highest prevalence of 8.4%.

### 3.2 Selected contextual factors and indicators

For AdiRaum, contextual factors were selected from the areas of settlement structure, sociodemographics or physical activity and food environment, and corresponding indicators were identified (see Table 1).

Data were not available for all context-related indicators for the five years of enrolment. For example, for the districts of Berlin the indicator ‘School leavers from general education schools: Proportion without secondary school certificate (in %)’ is only available for 2018 and 2019 in the Regionaldatenbank [21]. Moreover, the indicators that could be differentiated by age group differed in part with respect to their age limits (0–6 years, 3–5 years, or <6 years). For cost, time, and resource reasons within AdiRaum, the age reference of the corresponding indicator was retained and no uniform age limits were produced through special evaluations.

For visualisation of the results in the Tableau dashboard, the indicators had to be categorised. This was mostly done based on previously established stratifications of indicators of the Deutschlandatlas [24], AdiMon [20] and the Ländermonitor Frühkindliche Bildungssysteme of Bertelsmann Stiftung [25]. For five indicators, no corresponding reference was found in an internet search. In these cases, the median served as a threshold to form two categories (much/little). No geospatial indicators could be reflected for merged counties of Bavaria, as no corresponding data were available.

The selected indicators (Table 1) were linked to the SEE data set via the county or district name.

### 3.3 Visualisation in the Dashboard

**Figure 2:**
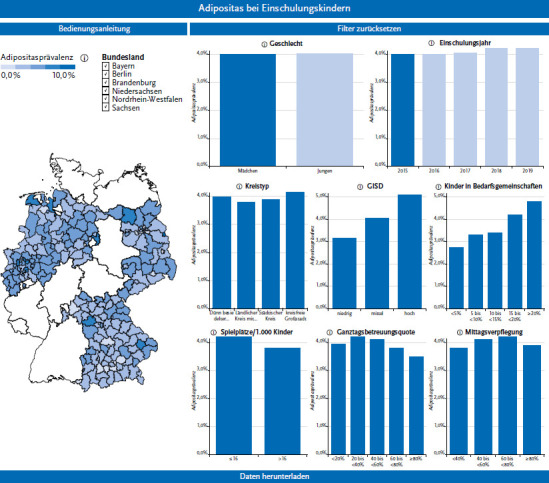
Dashboard of AdiRaum on obesity prevalences of children of school enrolment-age at county level (screenshot) Source: own diagram

For visualisation of the data, a dashboard was created with TableauTM software. In the dashboard the prevalence of obesity by rural county, independent city or district is shown on a map of Germany (Figure 2). The prevalence is shown colour-coded in five different categories (ranging from 0 to ≥8%). Individual counties can be selected manually. A pop-up window displays the name of the respective county, the number of children examined there, and the obesity prevalence. In addition to the map, column charts show the prevalence stratified by gender and year of enrolment, as well as by selected indicators. By setting filters, the prevalences shown in the map can be narrowed down by county according to these characteristics. Multiple filters can be selected. By selecting indicator categories, it is possible to display the prevalences of districts that are similar in terms of settlement structure, sociodemographics or physical activity and food environment on the map of Germany. In the current version of the dashboard, only one year can be stored for each indicator. It is possible to download selected prevalences.

Prevalences for different settlement-structural county types (sparsely populated rural county, rural county with initial densification, urban county, independent large city) or the degree of regional deprivation according to the German Index of Socioeconomic Deprivation (GISD) are displayed by selecting the appropriate filters. For example, the obesity prevalences of the 2015 school enrolment cohort ranged for sparsely populated rural counties from 2.1% to 8.4%, and from 1.5% to 6.7% for independent cities. The average obesity prevalence in 2015 was 3.3% in counties with low levels of deprivation, 4% in counties with medium levels of deprivation, and 5.2% in highly deprived counties. The release of the dashboard is currently being piloted and planned for mid-2023.

## 4. Discussion

In AdiRaum, it was possible for the first time to merge aggregated data from school entry examinations of several federal states using obesity prevalences on a small-scale level for exemplary purposes. In cooperation with representatives of health reporting departments of the states, the obesity prevalences in years of enrolment 2015 to 2019 of six federal states were merged at county or district level (if available), linked to context-related indicators, and interactively visualised in a dashboard.

AdiRaum piloted the merging of aggregated SEE data on county level from six federal states, thus went beyond mere data research on prevalences at the federal state level [11]. The feedback received from the representatives of the federal states showed that the data transfer was possible with little effort due to the agreements made beforehand and the provision of an Excel template. This made it possible to deliver the data to the RKI on time. Thus, data from complete age cohorts were available for these federal states.

Upon consolidation of the small-scale data on obesity from the SEE of various federal states, the indexing and linking of context-related indicators, and the presentation of the results in an interactive dashboard, findings are available that are discussed below in exemplary manner. On this basis requirements for a future continuation and expansion of the project are outlined before the selected method is discussed with regard to its limitations and strengths.

## Indexing and use of context-related indicators

The majority of the context-related indicators were freely available in public data sources or could be obtained free of charge on request (sports club memberships) from the regional sports confederations and the Statistical Office Berlin-Brandenburg. Nevertheless, the effort required to index the indicators was not negligible. Indicators were available for different – albeit similar – age groups. For future use, special evaluations should be commissioned in order to align all indicators with regard to the parameter of age.

A major difficulty in the search for data sources was the selected regional depth (county level), at which data gaps were identified. For example, relevant indicators for obesity had to be excluded for Berlin districts due to a lack of availability at the small-scale level.

In order to better investigate the context of the living environment of children and adults, environmental indicators with health relevance, such as walkability and bikeability, should be established at the small-scale level. The challenge here is to define and reflect the structural properties for these indicators [2].

## Visualisation

Using the TableauTM software, a user-friendly visualisation of obesity prevalences on county level was developed, which provided a comparison between counties with similar settlement structure or sociodemographics. In the pilot project, the interface was limited to a few functions, but these can be expanded in the future. Modifications to the dashboard would then also allow for the display of time series of indicators, changes in dynamic indicators, such as the proportion of children in communities in need, and their correlations with obesity prevalences. A publicly accessible version of the dashboard is a prerequisite for widespread use.

## Limitations

In Germany the implementation of the SEE is mandatory nationwide, but determined federally through state laws and supplementary framework agreements. For this reason, the survey methods and the surveyed parameters used to determine the developmental status of children differ considerably between the federal states. There are also differences in the cut-off dates for school enrolment and in the examination period covered by the SEE. As a result, the age of the children at the time of the SEE varies between federal states.

An exception is the measurement of height and weight, which is done in all federal states participating in AdiRaum. The SEE data are collected in a standardised manner, but inaccuracies in measurements and rounding by medical staff cannot be completely excluded. In the course of the project, it turned out that height and weight are measured in the SEE by the Child and Adolescent Health Services or through examinations for early detection of diseases (so called U9). This is done by paediatricians between 60th and 64th month of life [26]. This limits the comparability of the data and must be considered in the interpretation, as the implementation of the U9 is not standardised.

Interpreting the regional obesity prevalences and linking them to context-related indicators, it must also be kept in mind that the indicators at county level do not allow direct conclusions to be drawn, for example, about the actual residential environment or the socioeconomic background of the children, since the linked data are aggregated and not individual data. The regional diversity for the selected indicator within a county may be extensive. Based on the current availability of data for the indicators a differentiated consideration of the prevalences at municipality level cannot be realised by the RKI, but possibly by the counties and municipalities.

Although the period of the SEE data from 2015 to 2019 was jointly agreed with the federal states not all counties had data available for the entire period. This could be attributed, among other things, to the different timeliness of data compilation in the public health service of the respective federal states. Therefore, the planning of future projects must consider that the data of current years, in particular, may not be available in all states at the same time.

## Strengths

The SEE data aggregated on a small-scale level and the linkage to context-related indicators allowed analyses of the SEE at county level and the comparison of similar counties across state borders for the first time. The transfer of the data to the RKI and the indexing for public health research were successfully tested. In addition, the utilisation of the SEE data is a resource-saving option for a continuous, nationwide monitoring. The data represent a full survey of an, albeit limited, age group and are thus indispensable for assessing various aspects of child health. Among other aspects, the data can potentially be used for further research on the correlations between socioeconomic background and health of preschool children.

## Outlook

Based on the visualisations in the dashboard and in addition to the brief presentation of results in this article, we plan to conduct further analyses and to publish the results together with the project partners. The focus is on the question of what role settlement structure and sociodemographic factors play in the differences in the prevalence of obesity among children of school enrolment-age at county level.

The participating federal states have expressed great interest in continuing the cooperation after completion of the project period and additional states have indicated interest in participating in a future project. An extension of the project to other federal states would bring the data base close to a nationwide full survey allowing living environment indicators to be mapped on a nationwide level. However, it can be assumed that in this case the diversity of the survey methods would increase, which would make the interpretation of the data more difficult. In the future the nationwide health monitoring could be extended to additional parameters of the SEE, provided that the data are collected in a uniform or comparable manner. In the pilot project, the analyses were focused on the prevalence of obesity. A future expansion to overweight – as a preliminary stage of obesity – or also underweight would be possible in this age group.

The SEE data are highly relevant for public health beyond the individual medical benefit. They are suitable to serve as a data basis for analysis of needs at county and district level and can be used in the future for targeted planning, implementation and evaluation of prevention measures and for detailed public health research on questions at population level. In this context, it should be examined whether uniform indicators for further parameters of the SEE can be developed in the future.

## Key statements

- In the SEE of the federal states children’s height and weight are collected in a standardised manner.
- Obesity prevalence data at county level are available from the SEE.
- The transfer and aggregation of the SEE data were successfully tested in AdiRaum.
- Linking the data to small-scale indicators enables regional analyses of obesity prevalence and cross-state comparisons of counties with similar settlement structure and sociodemographics.
- The SEE are a data base that should be used as part of a continuous national health monitoring of children.

## Figures and Tables

**Table 1:** Selection of context-related indicators of AdiRaum Source: own table

Subject area/Contextual factor	Indicator	Data source	Data access	Categories
**Settlement structure**				
**Degree of urbanisation**	Settlement-structural county types	Federal Institute for Building, Urban Affairs, and Spatial Development	Indicators and Maps of Spatial and Urban Development	Sparsely populated rural district
				Rural district with initial densification
				Urban county
				Independent city
**Regional deprivation**	German Index of Socioeconomic Deprivation (GISD)	Robert Koch Institute	Robert Koch Institute	low
				medium
				high
**Sociodemographics**				
**Poverty**	Proportion of children in communities in need (SGB II) among children of the same age (in %)^1^	Statistics of the Federal Employment Agency	Federal Employment Agency	<5%
				5–<10%
				10–<15%
				15–<20%
				≥20%
**Education**	School leavers from general education schools: Proportion without lower secondary school-leaving certificate (in %)^2^	Statistics on the schools of general education	Regionaldatenbank	≤5.8%
				>5.8%
**Physical activity and food environment**				
**Kindergarten**	Proportion of children in all-day care (>7h/day) among children of the same age (in %)^3^	Statistics of child and youth welfare	Regionaldatenbank; Statistical Office Berlin-Brandenburg	<20%
				20-<40%
				40-<60%
				60-<80%
				≥80%
	Proportion of children receiving lunch among all children in care (in %)^1^	Statistical Offices of the Federal States	German Federal Statistical Office	<40%
				40-<60%
				60-<80%
				≥80%
**Physical activity areas**	Number of playgrounds per 1,000 children ^2,4^	Open Street Map	Geo-Fabrik	≤16
				>16
	Number of sports facilities per 1,000 children ^2,4^	Federal Agency for Cartography and Geodesy	Digital Basic-Landscape Model (Basic DLM)	≤11
				>11
	Green spaces (ha) per 1,000 children ^2,4^	German Federal Statistical Office; Statistical Offices of the federal states	Regionaldatenbank	≤41
				>41
**Sports activity**	Number of memberships in sports clubs per 100 children ^4,5^	Regional sports confederations; Statistical Office Berlin-Brandenburg	Regional sports confederations; Statistical Office Berlin-Brandenburg	<30
				30–<40
				40–<50
				50–<60
				≥60
**Miscellaneous**				
**Medical care**	Number of paediatricians per 1,000 children ^2,4^	National Association of Statutory Health Insurance Physicians	Indicators and Maps of Spatial and Urban Development	≤0.4
				>0.4

^1^ refers to children between 0 and 5 years of age

^2^ Classification on the basis of the median as ‘much’ (value is above the median) or ‘little’ (value is below the median)

^3^ refers to children between 3 and 5 years of age

^4^ refers to children between 0 and 6 years of age

^5^ For Bavaria: refers to children between 0 and 5 years of age

SGB II = Second Book of the German Social Code
